# The Effect of Design Rainfall Patterns on Urban Flooding Based on the Chicago Method

**DOI:** 10.3390/ijerph20054245

**Published:** 2023-02-27

**Authors:** Jian Chen, Yaowei Li, Changhui Zhang

**Affiliations:** Water Conservancy College, North China University of Water Resources and Electric Power, Zhengzhou 450046, China

**Keywords:** design rainfall, Chicago rain-type method, urban flooding, numerical models

## Abstract

Design rainfall is the basis for deriving design floods in areas where rainfall data are lacking and has a significant impact on the construction of water engineering facilities and municipal engineering designs. The Chicago rainfall pattern method has great applicability for urban short-duration design rainfall. In order to analyze the influence of design storm rainfall patterns on urban flooding, numerical models of hydrological and hydrodynamic processes were applied to simulate design rainfall with different recurrence periods and different rain peaks and were also used to compare and analyze the total amount of water accumulation and inundation extent by taking the central city of Zhoukou as an example. The results show that when the design rainfall recurrence period is less than 20 years, the total volume and inundation extent of waterlogging in design rainfall with a smaller peak ratio is larger. When the return period is greater than 20 years, the pattern is reversed. However, as the return period grows, the difference in peak inundation volume due to different peak rainfall amounts decreases. This study has certain guiding significance for urban flood forecasting and early warning efforts.

## 1. Introduction

According to the Sixth Assessment Report issued by the Intergovernmental Panel on Climate Change (IPCC) [1], the global average temperature in the first two decades of the 21st century was 0.99 °C higher than the 1850–1900 average. Global warming, the increased probability of extreme rainfall [2,3], accelerated urbanization, and an increased amount of impervious surfaces have made urban flooding frequent [4]. For example, on 2012 “7.21”, Beijing’s extraordinarily heavy rainfall caused 1.602 million people to be affected and led to economic losses amounting to USD 169 million. On 2014 “7.27”, Hefei’s heavy rainfall event caused nearly one million people to be affected and economic losses amounting to USD 174 million. In July 2021, 14,531,600 people in the province of Henan were affected by the extraordinarily heavy rainfall in Zhengzhou, with a direct economic loss of USD 16.61 billion [5]. Waterlogging has become an important issue in the development of urbanization.

In recent years, the amount of research into urban flooding has gradually increased. For example, Xu et al. [6] simulated the flooding process of a 500-year rainstorm in Jiangbei City and Fuzhou City. Li et al. [7] simulated the inundation situation under different recurrence periods based on the SWMM model to determine the flooding risk zone. Li et al.’s [8] model simulated the role of rain gardens in regulating runoff under different precipitation intensities. However, there is another important factor in the effect of rainfall on flooding: the rainfall pattern. Rainfall pattern describes the distribution of precipitation intensity over time and directly influences the magnitude and location of rainfall peaks while also being directly related to the severity of urban flooding. Therefore, it is important to qualitatively and quantitatively analyze the influence of rainfall patterns on urban flooding. In recent years, there have been more and more studies on rain pattern characteristics. For example, Liu et al. [9] statistically analyzed the rainfall pattern characteristics of Hangzhou; Wang et al. [10] used the Chicago rain pattern method and the Pilgrim and Cordery rain pattern method to derive the design storm rain pattern distribution in Kunshan; Tang et al. [11] analyzed the amplification methods for the different rain patterns of measured rainstorms; and Zhao et al. [12] used the fuzzy identification method to classify the rainfall patterns in Beijing. Most of the above studies studied urban flooding in terms of different rain intensities and rain volumes.

In the study and analysis of flooding, most of the typical rainfall types in the region are also used. For example, Wang [13] conducted a simulation of flooding in a bimodal rainfall-type urban drainage and waterlogging problem with a short calendar bimodal rainfall design storm for each recurrence period. Li et al. [8] studied the reduction effect of rain gardens on storm runoff by choosing a typical Chicago rain pattern in Xi’an, and also chose multiple rain patterns to avoid the specificity of the storm in the text. Forestieri et al. [14] used a hydrological model to simulate the flood-causing process of different designed storm rainfall patterns in the study area of the Sicily Basin in order to derive the disaster-causing critical rainfall warning mechanism applicable to various rain patterns. Pedrozo-Acuna et al. [15] studied and analyzed flooding caused by historical measured rainstorms of different rainfall patterns under each return period in Tabasco, Mexico, and came up with a scheme to identify flood-prone sites under each rain pattern and countermeasures. Additionally, Tang et al. [16] monitored the flow in and out of rain gardens under all measured rainfall conditions over a 4-year period to analyze their runoff reduction effects. However, none of the above studies paid attention to the differences in flooding hazards under different storm rainfall patterns.

However, in addition to rain intensity and rainfall, their temporal distribution also has a great impact on urban flooding. The design storm is the likely local rainfall that meets the specified design criteria and is used to derive the design flood for flood prevention purposes. The design storm rainfall pattern is the rainfall process (distribution of rainfall intensity over time) of the design storm, i.e., the temporal distribution of the design storm [17].

The Chicago rain pattern method is the distribution of frequency for the storm intensity formula, and the rainfall in any calendar period in the derived design storm rain pattern is equal to the design rainfall; thus, the calculated flood flow is relatively stable and easy to use. Therefore, this paper takes the central urban area of Zhoukou City as an example and is based on the MIKE model. Scenario projection of the waterlogging process, the systematic analysis of urban flooding inundation in the central urban area of Zhoukou City under different design rainfall rain patterns in terms of inundation area and inundation depth, and the analysis of the response characteristics of urban flooding to rainfall rain patterns were conducted with a view to providing reference information for urban flooding early warning and forecasting, disaster prevention and control, etc.

## 2. Materials and Methods

### 2.1. Overview of the Study Area

**Figure 1:**
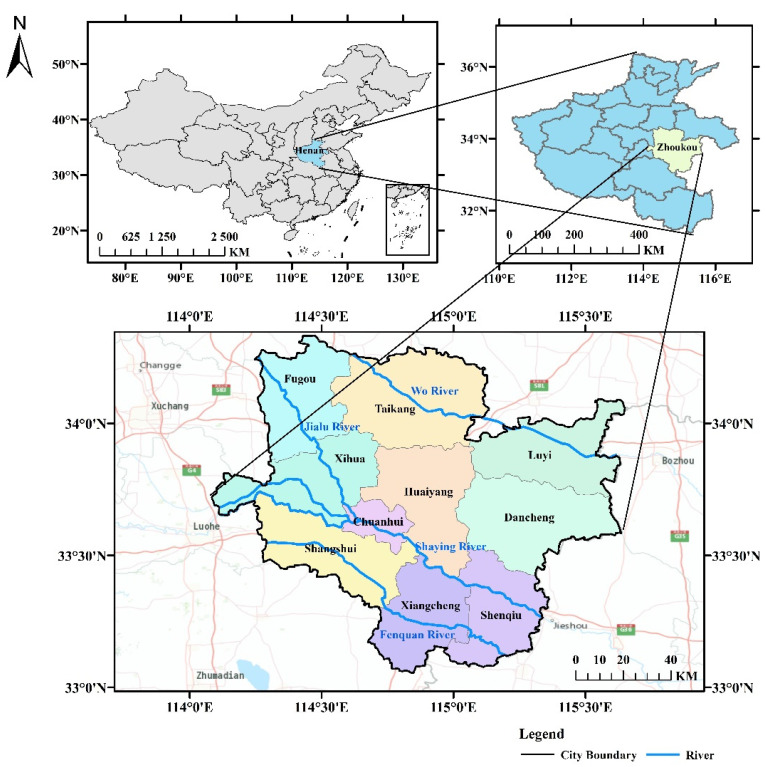
Zhoukou City location map.

Zhoukou is located in the East Henan Plain, southeast of Henan Province. The geographical coordinates are 33°03′–34°20′ N and 114°05′–115°39′ E and the city is 135 km wide from north to south and 140 km long from east to west [5]. A location map of Zhoukou City is shown in Figure 1. At the end of 2021, Zhoukou had a resident population of 8.8530 million, with a total area of 323km^2^ and a population of about 1.02 million in the central part of the city. The city’s annual gross domestic product is RMB 34.962 billion, up 6.3% year-on-year. Zhoukou City receives rainfall mostly in the form of heavy rainfall. This rainfall type produces flow at a fast rate, which easily causes rainwater runoff with high peak flow, thus generating high pressure on the drainage system and easily causing waterlogging in the city. An analysis of current urban built-up land in Zhoukou City shows that the proportion of hardened underlayment is high, and the indicators for green areas and squares are significantly low, accounting for about 4% of total urban land (less than 5m^2^ per capita). City planning in Zhoukou requires that green areas and squares occupy no less than 10 m^2^ per capita [18].

### 2.2. Data

#### 2.2.1. Lower Bedding Surface Data

**Figure 2:**
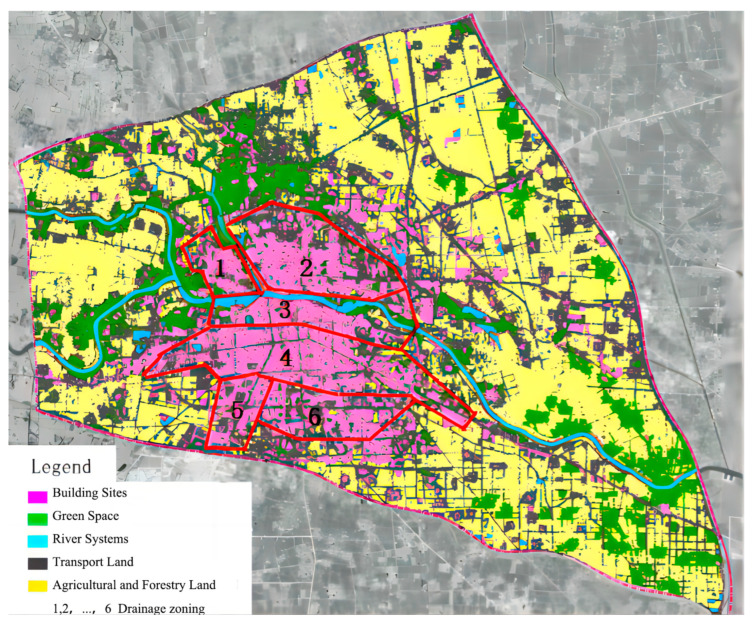
Map of the sub-bedding surface of the central city of Zhoukou.

Urban rainfall surface runoff is mainly influenced by the intensity of rainfall and the subsurface. Using a combination of field surveys, large-scale topographic maps, and high-resolution remote sensing images to analyze the subsurface within the planning area and comprehensively analyze the types of water bodies, grass, woods, bare soil, roads, squares, and roofs and paving in small areas allowed for subsurfaces in the central city of Zhoukou to be classified into five major categories: river systems, green space, transport land, building sites, and agricultural and forestry land. For details, see Figure 2 and Table 1.

#### 2.2.2. Design Storm Calculations

##### Storm Intensity Formula

The equation used to calculate the intensity of rainstorms in the central city of Zhoukou is presented below [19].

$$i=\frac{q}{t}=\frac{a*(1+c*\mathrm{LgP})}{167*(t+b{)}^{n}}\mathrm{mm}/\min$$

Mean absolute standard deviation: 0.039mm/min; mean relative standard deviation: 4.92%, where a = 3032.247, c = 0.828, b = 21.405, n = 0.755, and q is the intensity of the storm (L/(s − h m^2^)). i indicates precipitation (mm). t is rainfall duration (min) and takes values from 5 to 180 min. p is the recurrence period (a) and takes values from 1 to 100a.

##### Design Storm Extrapolation

The Chicago rain pattern method is also known as the Keifer and Chu method. The Chicago rain pattern model [20] is derived from the storm intensity equation, and the location of the rain peak (integrated rainfall coefficient r) is determined from statistics of previous years’ rainstorm events. The Chicago rain pattern is applicable to urban areas, and the design rainfall ephemeris of around 3 h is the most accurate. Using the Chicago rain pattern as the design rain pattern for this occasion, the parameters of the storm intensity formula are initially determined. Rain intensity of the rising section (pre-peak) and falling section (post-peak) is then calculated separately using the formulae listed below for certain minute moments, and the instantaneous rainfall process line is finally obtained.

$$i_{b}=\frac{a[\frac{(1-c)\;t_{b}}{r}+b]}{{\left(\frac{t_{b}}{r}+b\right)}^{c+1}}$$

$$i_{a}=\frac{a[\frac{(1-c)\;t_{a}}{1-r}+b]}{{\left(\frac{t_{a}}{1-r}+b\right)}^{c+1}}$$

where *a*, *b*, and *c* have the same meaning as in the formula for storm intensity, *r* is the moment of peak rainfall, *b* is pre-peak rain intensity, *i*_*a*_ is post-peak rain intensity, *t*_*b*_ is the pre-peak rainfall calendar time, and *t*_*a*_ is the post-peak rainfall calendar time.

**Figure 3:**
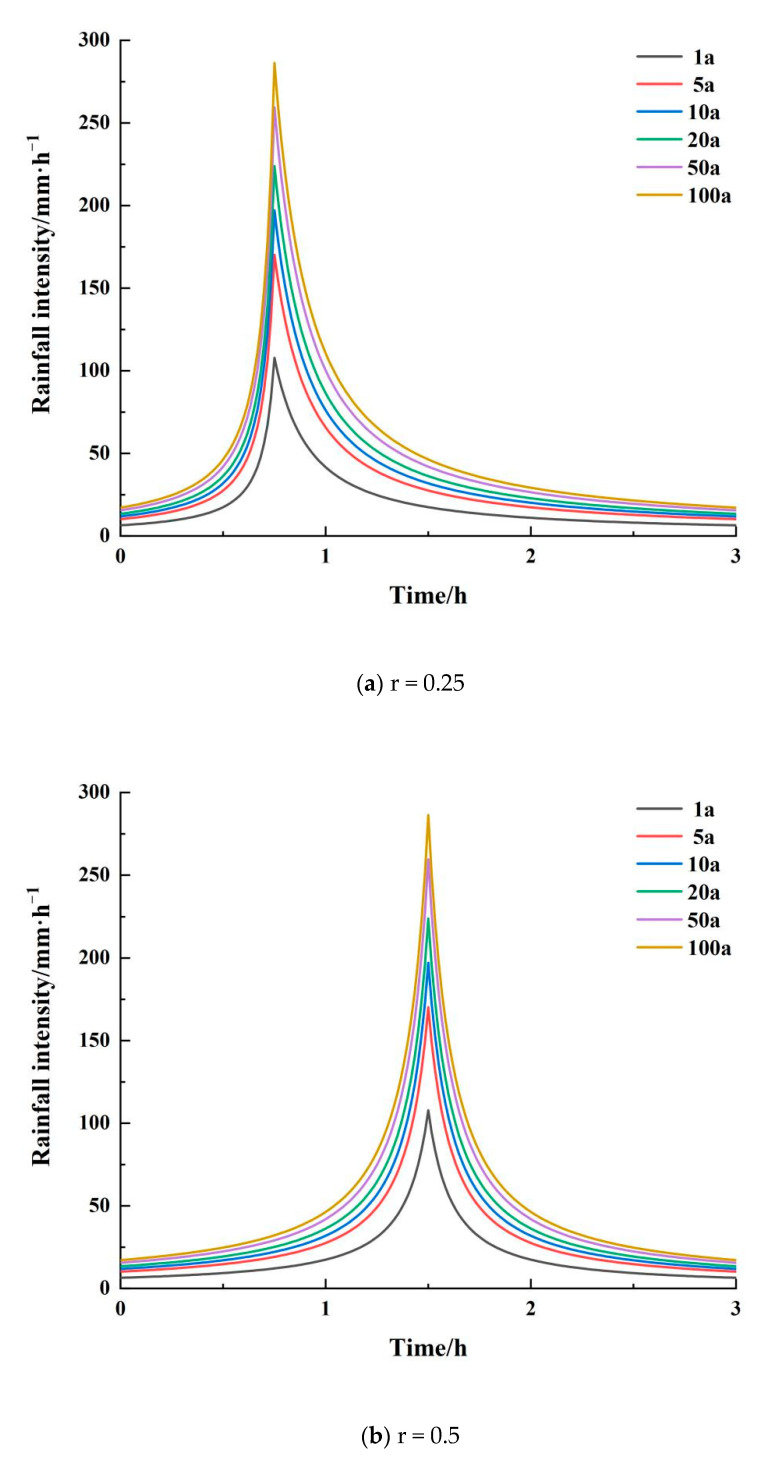

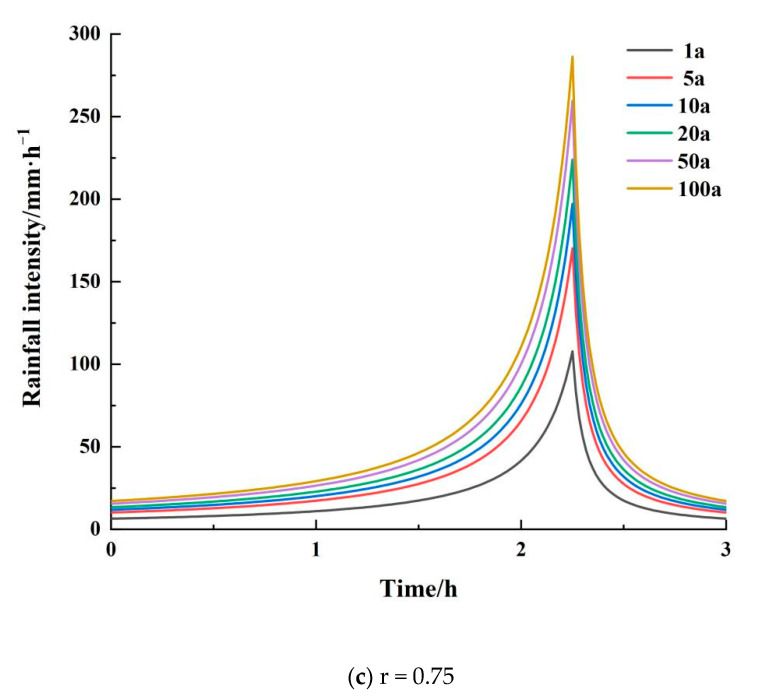
Chicago rain pattern.

The values of the relevant parameters in the Chicago rain pattern are all consistent with the rainstorm intensity formula, and only the rain peak position r is a new parameter. According to the analysis of the multi-year average rainfall data of Zhoukou City from 1966 to 2016, it can be seen that the central city of Zhoukou City receives mostly single-peak rainfall, with a comprehensive peak ratio of r = 0.4. In order to explore the effect of different peak ratios on internal flooding, the Chicago rain type was chosen as the design rainfall and three peak ratios (r = 0.25, r = 0.5, and r = 0.75) for characteristic rain types were chosen. A total of 18 design rainfall scenarios were explored, as shown in Figure 3.

### 2.3. Model Construction and Validation

#### 2.3.1. Model Construction

The MIKE FLOOD platform integrates a one-dimensional stormwater network model (MIKE URBAN) and a two-dimensional surface diffuse flow model (MIKE21) to build a coupled urban flooding model, and MIKE URBAN is a professional simulation software for urban drainage networks that was developed by the Danish Hydraulic Institute (DHI), which mainly consists of a rainfall infiltration module and a pipe confluence module [21]. MIKE 21 can be coupled with MIKE to simulate flooding in two dimensions. Studies have shown that the modelling efficiency of MIKE URBAN is higher, and the modelling effect based on user-defined time area curves is higher than that of SWMM [22]. For example, Luan et al. [23] evaluated the drainage capacity of a pipe network system using the SWMM-MIKE 11 coupled model and verified the value of the model in urban flood risk assessment. Lu et al. [24] performed rate determination of the MIKE FLOOD model and verified the effectiveness of the model in a simulation of flooding in the Qinghe River Basin in Beijing. Zhang et al. [25] constructed a coupled urban flooding model based on the MIKE FLOOD platform and simulated flood control and drainage in new urban areas by combining LID measures. Luan et al. [26] simulated flooding scenarios and flood risk assessment in typical areas based on the MIKE FLOOD platform. Kong et al. [27] developed a coupled SWMM-MIKE 11 model to complement each of the models and used them as a tool to promote the construction of sponge city rivers.

The constructed 1D river model includes 12 rivers with 682 cross sections. The two-dimensional surface diffuse flow model simulates an area of about 145.75 km^2^ using a rectangular grid with a grid size of 2 m × 2 m, a grid number of 3.64 × 10^7^, and a grid area of 4 m^2^. The pipe network model consists of 11,029 inspection wells and 11 pumping stations. The above three models are dynamically coupled in MIKE FLOOD to construct a coupled urban flooding model.

#### 2.3.2. Model Validation

**Figure 4:**
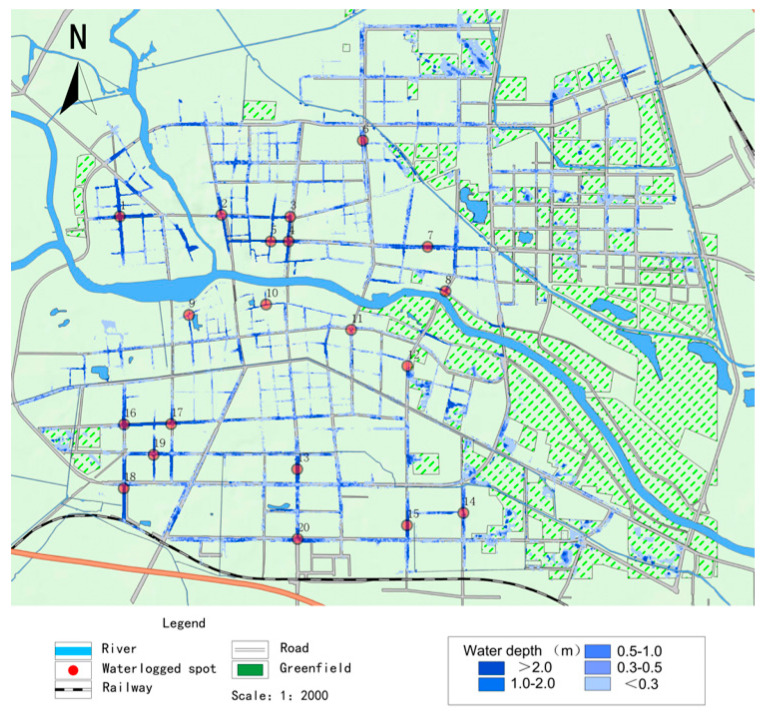
Distribution of major waterlogged sites on 20 July 2021.

Using 20 July 2021 in Zhoukou City as an example, 20 major waterlogging points in the central city were selected, and the distribution of waterlogging points is shown in Figure 4. The measured maximum ponding depth data were compared with the maximum ponding depth simulated by the numerical model, as shown in Table 2. It can be seen that the simulated and measured values are close to each other, with the difference being within 5cm, which is a small error. This indicates that the numerical model developed is close to reality.

## 3. Results and Discussion

### 3.1. Total Waterlogging Analysis

**Figure 5:**
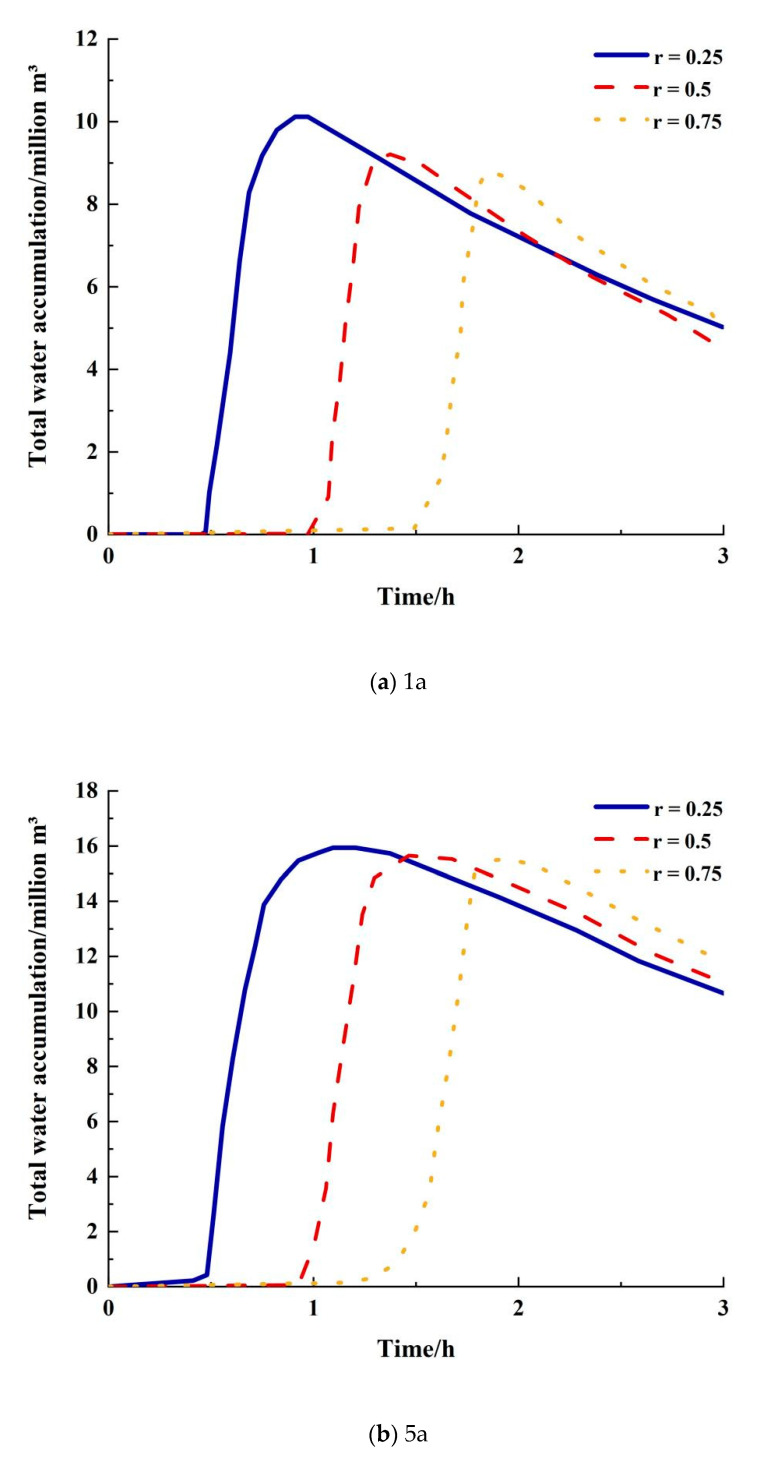

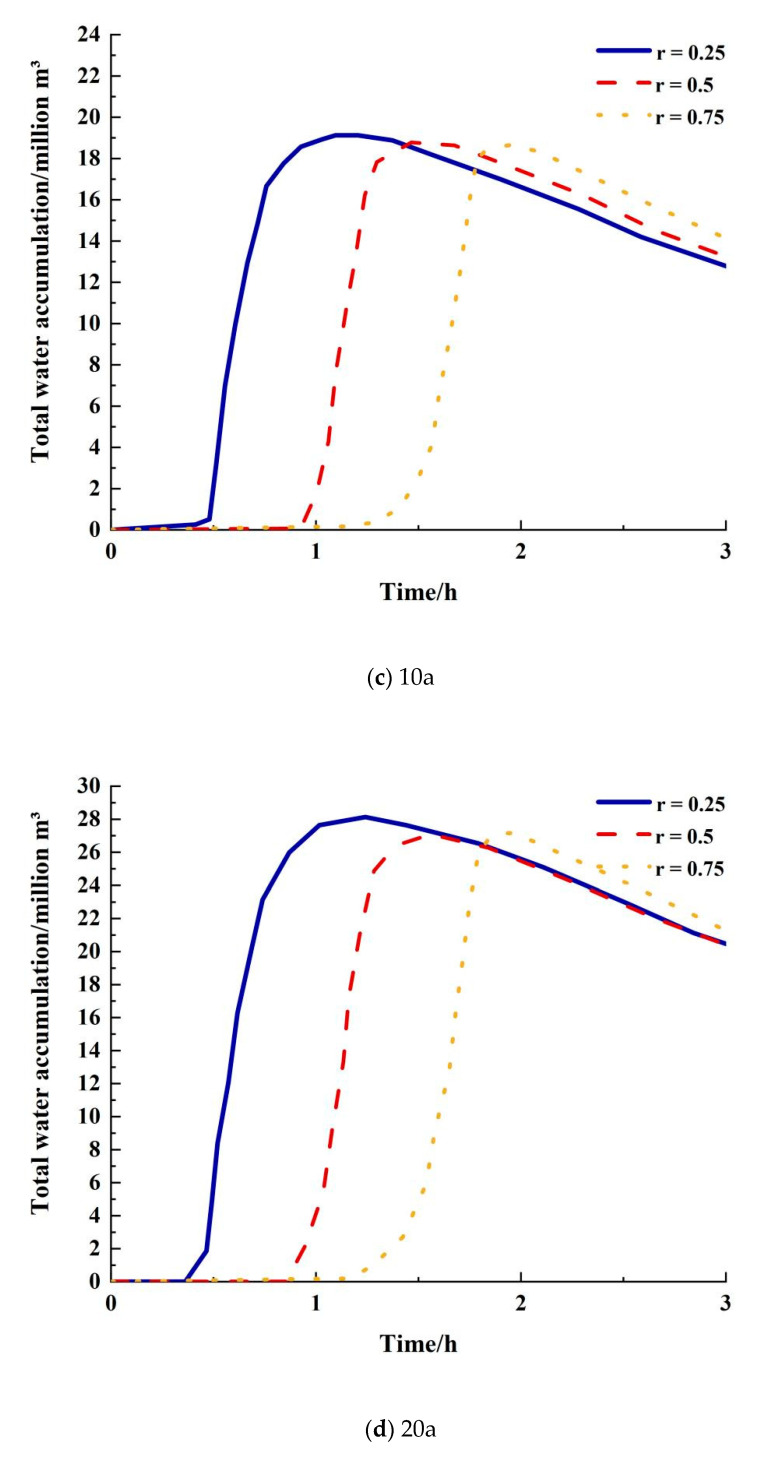

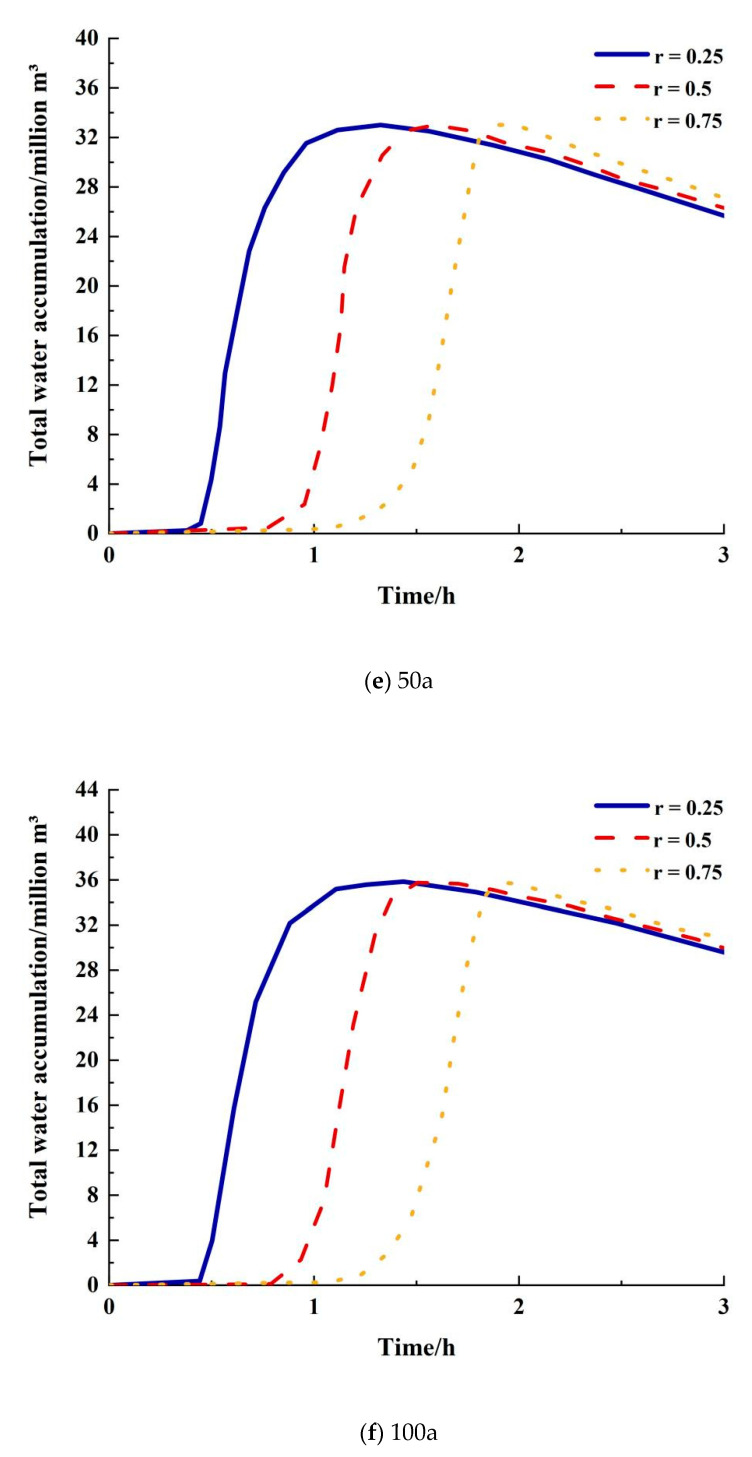
Variation in total water accumulation by rainfall type for different recurrence periods.

The waterlogging situation in the study area can be expressed in terms of the total amount of waterlogging. The total amount of water accumulated at each ponding point in the study area was superimposed to obtain the total amount of water accumulated in the area, and the process of calculating the total amount of water accumulated for each rainfall type at different return periods is shown in Figure 5. A comparison of the peak amounts of water accumulated at different return periods is shown in Table 3. As can be seen from Figure 5 and Table 3, when the return period is less than 20 years, the Chicago design rainstorm with a peak ratio of r = 0.25 is the most severe. For example, for a return period of 10 years, the total peak ponding volume for a peak ratio of r = 0.5 is 187,800 m^3^, a reduction of 1287 m^3^ from the peak ratio of r = 0.25; the total peak ponding volume for a peak ratio of r = 0.75 is 186,400 m^3^, a reduction of 1300 m^3^ from the peak ratio of r = 0.5. When the return period is higher than 20 years, a peak ratio of r = 0.75 produces a higher peak inundation than a single-peaked rainfall with r = 0.25. However, as the intensity of precipitation increases, the difference in peak inundation due to the difference in peak coefficients decreases. For example, for a 50-year return period, a rainfall with a peak ratio of r = 0.75 produces 4010 m^3^ more rainfall than a rainfall with r = 0.25. For a 100-year return period, a peak ratio of r = 0.75 provides 2962 m^3^ more rainfall than a peak ratio of r = 0.25.

It can be seen that, for design storms with different peak ratios, the more advanced the rainfall peak the more severe the inundation is when the rainfall return period is less than 20 years, and the more lagging the rainfall peak the more severe the inundation is when the rainfall return period is more than 20 years; however, as the return period increases, the difference in peak inundation from different types of rainfall decreases.

### 3.2. Inundation Extent Analysis

**Figure 6:**
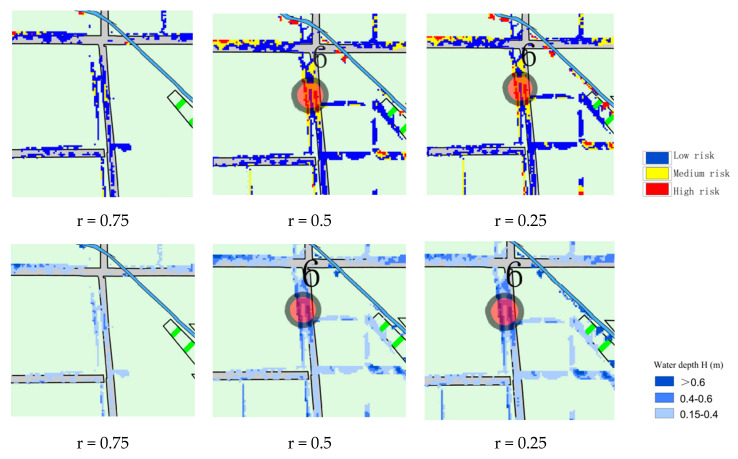
Map of the risk of local flooding under different rainfall patterns for the 1 in 10 year event.

The inundation scenarios were extrapolated for the design rainfall, and Figure 6 illustrates the local inundation risk maps for each of the 10-year rainfall types. It can be seen that the peak ratio r = 0.25 has the largest inundation extent, r = 0.5 is the next largest, and r = 0.75 is the rain type that results in the smallest inundation extent.

In order to visualize the difference in inundation area due to different rainfall patterns, Table 4 shows the peak inundation area and the growth rate of the inundation area between adjacent return periods. It can be seen that as the recurrence period increases, the inundated area increases. As the intensity of rainfall increases, low-lying areas are covered by floodwater and higher areas around buildings are gradually inundated. In the 100-year rainfall, excluding the areas covered by buildings, basically all of them are inundated; thus, the growth trend of inundated areas gradually becomes slower. For the same rainfall return period, there are some differences in the peak inundation areas caused by different rainfall patterns. When the return period is less than 20 years, the inundation area produced by rainfall with r = 0.25 is greater than that produced by rainfall with r = 0.75; when the return period is greater than 20 years, the inundation area produced by rainfall with r = 0.25 is lower than that produced by rainfall with r = 0.75. Except for the return period of 20 years, the ponded area for r = 0.5 is greater than r = 0.25 and r = 0.75.

## 4. Conclusions

(1) The design rain pattern based on the Chicago method is a guide to urban flood forecasting and warning systems through the extrapolation of internal flooding scenarios.

(2) For different peak ratios of rainfall (when the rainfall return period is less than 20 years), the more advanced the rainfall peak, the more severe the inundation produced. When the rainfall return period is higher than 20 years, the further back the rainfall peak is, the more severe the inundation produced. As the return period increases, the difference between the peaks of inundation caused by different types of rainfall decreases.

(3) By extrapolating scenarios of the inundation process, it can be seen that the peak inundation area due to the design rainfall of different rainfall types increases with the increase in the return period, and the growth trend becomes gradually slower. For the same return period, there are differences in the peak inundation areas caused by different rainfall types. r = 0.25 yields a higher inundation extent and a larger peak inundation amount than r = 0.75 when the return period is less than 20 years; the situation is reversed for return periods exceeding 20 years. In terms of peak inundation amounts, r = 0.5 is between r = 0.25 and r = 0.75. In terms of the inundation area however, r = 0.5 is largest.

This study can help more reasonable and effective flood control work to be carried out and has some application prospects. It also has certain guiding significance for urban flood forecasting.

## Figures and Tables

**Table 1:** Surface types (lower bedding surface) in the central city of Zhoukou.

Serial Number	Drainage Zoning	Surface Type (Subsurface) Area Statistics (ha)					
		River Systems	Green Space	Transport Land	Building Sites	Agricultural and Forestry Land	Total
1	Jalu River Drainage Subdivision	68.66	0	47.1	293.81	307.75	717.32
2	Puddle Wash Drainage Subdivision	32.28	57.17	108.21	705.24	1065.94	1968.84
3	Sandy River Drainage Subdivision	38.39	1.10	72.44	578.28	56.12	746.33
4	Traffic Main Drainage Subdivision	119.13	15.94	264.55	1088.98	684.98	2173.58
5	Yang Brain Dry Drainage Subdivision	2.40	31.58	51.28	342.71	147.27	575.24
6	Canal Drainage Subdivision	8.72	0	52.41	533.98	268.34	863.45

**Table 2:** Comparison of measured and numerically modelled ponding depths at inundated water points on 20 July 2021.

Serial Number	Location of Ponded Water	Measured Water Depth/cm	Simulated Water Depth/cm	Difference/cm
1	Bayi Road North	60	65	5
2	Junction of Qi Yi Road and Da Qing Road	40	42	2
3	Near Gongnong Road and Yuxin Street	55	58	3
4	Intersection of Construction Road and Yinzhu Road	30	31	1
5	Area around Sanlian Hang	80	76	−4
6	West Street Community	80	84	4
7	Jianxi Street area	60	56	6
8	Area near Anju Road	40	45	5
9	Fumin Road South area	40	43	3
10	Hanyang Road South area	40	41	1
11	Jiancai Road area	40	38	−2
12	Gao Zhuang Community	40	42	2
13	Wenchang road	60	58	−2
14	Bayi Road and Construction	60	63	3
15	Zhou Shi gate	40	42	2
16	Civil Service District A	60	63	3
17	Yuxin Street East	40	42	2
18	Tai Hao Road, Zijingcheng District	35	39	4
19	Junction of Chaoyang Road	30	34	4
20	Tai Hao Road, Wu Yi junction	30	36	6

**Table 3:** Comparison of peak water accumulation at different return periods.

Reproduction Period	V (r = 0.5) − V (r = 0.25)/m³	V (r = 0.75) − V (r = 0.5)/m³
1	−2627	−3100
5	−1543	−2500
10	−1287	−1300
20	430	820
50	1150	2860
100	980	1982

**Table 4:** Peak inundated area and growth rate under different rainfall scenarios.

Reproduction Period/a	Peak Flooded Area/Million m²			Growth Rate/%		
	r = 0.25	r = 0.50	r = 0.75	r = 0.25	r = 0.50	r = 0.75
1	1064.1	1125.8	1023.5	0	0	0
5	1367.8	1442.5	1347.6	28.54	28.13	31.67
10	1555.4	1621.5	1532.7	13.72	12.41	13.74
20	1674.3	1670.3	1623.6	7.64	3.01	5.93
50	1853.2	1923.4	1877.5	10.68	15.15	15.64
100	2001.5	2040.5	2023.2	8.00	6.08	7.76

## Data Availability

Data and materials are available from the corresponding author upon request.
